# Predictors of Persistent Medically Unexplained Physical Symptoms: Findings From a General Population Study

**DOI:** 10.3389/fpsyt.2018.00613

**Published:** 2018-11-20

**Authors:** Jonna F. van Eck van der Sluijs, Margreet ten Have, Ron de Graaf, Cees A. Th. Rijnders, Harm W. J. van Marwijk, Christina M. van der Feltz-Cornelis

**Affiliations:** ^1^Top clinical Center for Body, Mind and Health, GGz Breburg, Tilburg, Netherlands; ^2^Department of Tranzo, Tilburg University, Tilburg, Netherlands; ^3^Netherlands Institute of Mental Health and Addiction, Utrecht, Netherlands; ^4^Department of Residency training, GGz Breburg, Tilburg, Netherlands; ^5^Division of Primary Care and Public Health, Brighton and Sussex Medical School, University of Brighton, Brighton, United Kingdom; ^6^Department of Health Sciences, Hull York Medical School, University of York, York, United Kingdom

**Keywords:** medically unexplained symptoms, prognosis, persistency, general population, course

## Abstract

**Objective:** To explore the persistency of Medically Unexplained Symptoms (MUS) and its prognostic factors in the general adult population. Knowledge of prognostic factors of MUS may indicate possible avenues for intervention development.

**Methods:** Data were derived from the Netherlands Mental Health Survey and Incidence Study-2 (NEMESIS-2), a nationally representative face-to-face cohort study among the Dutch general population aged 18–64 years. We selected subjects with MUS at baseline and who participated at follow-up (*N* = 324) and reassessed those subjects for having MUS at 3 year follow-up. Logistic regression analyses were used to determine risk factors for persistency of MUS.

**Results:** 36.4% of the subjects had persistent MUS at follow-up. In logistic regression analyses adjusted for sex and age, persistency of MUS was predicted by the number of comorbid chronic medical disorder(s), lower education, female sex, not having a paid job, parental psychopathology as well as lower functioning. In the logistic regression analysis in which all significant variables adjusted for sex and age were entered simultaneously, three variables predicted persistent MUS: parental psychopathology, the number of comorbid chronic medical disorder(s) and physical functioning, with odds ratios of 2.01 (1.20–3.38), 1.19 (1.01–1.40), and 0.99 (0.97–1.00), respectively.

**Conclusion:** In the adult general population, MUS were persistent in over one third of the subjects with MUS at baseline. Persistency was significantly predicted by parental psychopathology, number of comorbid chronic medical disorders, and physical functioning. These findings warrant further research into early intervention and treatment options for persons with an increased risk of persistent MUS.

## Introduction

### Background

Persons with Medically Unexplained physical Symptoms (MUS) are highly prevalent in health care settings (1), and in the general population (2, 3). MUS can be defined as physical symptoms for which a physician cannot find a sufficient explanation after proper medical examination (4–6). Persistent MUS can be severe and disabling (7–11), and lead to high health care use (12) and high costs (13). Persons with persistent MUS can be tempted to seek treatments that are not supported by scientific evidence(14). A specific focus on MUS is important, because treatment plans for MUS usually differ from treatment plans for chronic medical conditions. For physical symptoms explained by a chronic medical disorder, guidelines for a multitude of chronic medical disorders exist and can be used. For MUS, guidelines for treatment of MUS can be used (6, 15). Therefore, it is important to gain insight in predictors of persistent MUS, so that in an early stage persons with a high risk of having persistent MUS can get adequate treatment, such as collaboration with or referral to a physiotherapist or psychologist. We know little about the prognosis of persons with MUS in the general population and its determinants, as previous longitudinal research was mainly performed in selected groups, namely hospital or primary care patients. More precise estimates of how many persons with MUS -i.e., in the general population- suffer persistently from MUS, and what factors influence this persistency facilitate resource and care planning and may indicate possible avenues for future intervention development. This is needed, because such interventions can perhaps lead to a more favorable course of MUS and thereby reduce the heavy burden and impairment persons with persistent MUS can experience and they can help to possibly reduce costs.

Among patients with MUS in a general medical outpatient clinic, respectively 29 and 24% of the patients still had MUS at 11.6 and 15.2 months follow-up (16, 17). In another study in a general medical outpatient clinic with a mean follow-up of 61 weeks, 38% of the patients reported that their symptoms persisted (18). Among patients with MUS in a neurology outpatient clinic with 8 months follow-up, as much as 54% of the MUS persisted (19). Persistence of MUS was predicted by a longer duration of the MUS at baseline (18), higher number of (either explained or unexplained) physical symptoms at baseline (17, 18), and female sex (17).

In primary care, 51.2% of the MUS at baseline persisted during the course of 1 year (1). In over half of these patients the main MUS was pain. Significant predictors for persistent MUS after 1 year among these primary care patients with MUS were: negative life events, autonomic sensations (the experience of bodily symptoms), and the attribution of MUS to somatic disease -rather than psychological factors- by the patient (1). In another primary care study, 56.8% of the MUS persisted over a 2 year follow-up, with symptoms in multiple organs being a predictor of persistency (20). Another study found negative affect -which is closely associated with depression- to be an important determinant of persistent MUS, most probably because it contributes to symptom evolution as well as symptom severity, whereas positive affect has a beneficial effect on somatic symptom evolution (21). A systematic review concludes that because of a lack of rigorous empirical evidence, more research is needed to identify relevant prognostic factors for persistent MUS (10), as these are needed to develop effective interventions.

In the general population, even less is known about the course of MUS in adults. Research mainly focused on adolescents; it is not clear that MUS in adolescents and in adults are the same phenomenon (22). Possibly, higher percentages of persistency can be found in selected secondary or primary care patient groups with MUS compared to in the general population, because the former groups would be the more severe and chronic cases of MUS. Persistent MUS was found in only 4.1% of community adolescents with MUS at baseline (mean age 11.1 years) over a period of 5 years (mean age at reassessment 16.3 years) (23). In these adolescents, risk factors measured at baseline of the study for persistent MUS were female sex, presence of depressive symptoms, poor self-rated health, and parents‘report of a high number of MUS among their adolescent child (23). In another study it was found that subjects who score high (above the 90th percentile of the total score) on various symptoms, MUS tend to persist more often from childhood to adulthood when compared to low-scoring subjects (24). Perfectionism was also found to be associated with the persistence of MUS, this effect was significantly mediated by symptoms of anxiety and depression (25). Childhood adversities such as abuse and neglect increased the risk of diverse chronic physical conditions in later life (26); possibly this is also the case for MUS. In a study focusing on non-referred adolescents with somatoform disorder, 35.9% still met this diagnosis 15 months later (27). Predictors of persistency were female sex, comorbid depressive disorder, parental psychopathology, and number of negative life events.

To our knowledge, only the persistency and predictors of persistent medically unexplained *pain* symptoms (MUS-pain) have been studied in the adult general population (28). One third (33.6%) of MUS-pain was persistent, when measured over an 11 years interval. From the possible predictors measured at baseline, only female sex was a risk factor for persistence of MUS-pain. These results on MUS-pain cannot be generalized to all MUS, because MUS such as dizziness, overtiredness, nausea, and irritable bowel syndrome could have different risk factors for persistence compared to pain. If those risk factors can be influenced or treated, this is important information for the development of interventions. From a clinical perspective, a shorter follow-up course of a few years may be more relevant than a much longer period of time, and will possibly result in more risk-indicators than were found in the study of Leiknes and colleagues who only focused on MUS-pain over a period of 11 years (28).

The aim of this study is to estimate the persistence of MUS and explore prognostic factors among the adult general population. Prognostic factors can be categorized as demographic variables, vulnerability (e.g., trauma, negative life events), comorbid mental, and physical disorders, and mental and physical functioning. This study will add to the body of literature, given that the previous literature mainly focused on selected groups, i.e., patients seen by doctors [in primary care (1, 10, 21) or clinical setting (16–19)]. These selected patient groups comprise especially those persons with MUS with a high burden of MUS (29), and high costs (13). Insight in the course of MUS and its predicting factors in the general population is important, because it will tell us which MUS have a high risk of becoming persistent.

### Objective

Our objective is to estimate the persistency of MUS and to determine what factors influence this persistency in the adult general population. Based on previous findings, we hypothesize that female sex, childhood trauma, parental psychopathology, presence of negative life events, presence of common mental disorders, number of comorbid chronic medical disorder(s), and worse physical or general functioning–all measured at baseline- are associated with persistence of MUS measured at 3-year follow-up.

## Methods

### Study design

In this general population study, we examine the persistency of MUS and what factors are associated with persistence of MUS, over 3 years. We report our findings according to the STrengthening the Reporting of OBservational studies in Epidemiology (STROBE) statement (30).

### Setting and participants

Data were derived from the Netherlands Mental Health Survey and Incidence Study-2 (NEMESIS-2). Methods of NEMESIS-2 are described in details elsewhere (31, 32). Here, we give a short description, which has been published earlier (33). NEMESIS-2 was approved by a medical ethic Committee (METIGG). After being informed about the study, subjects provided written informed consent. For this paper, data were used from the first wave (T_0_), performed from November 2007 to July 2009, and the second wave (T_1_), performed from November 2010 to June 2012.

NEMESIS-2 is based on a multistage, stratified random sampling of households, with one respondent aged 18–64 years randomly selected in each household for a face-to-face interview. The interviews were conducted by professional, experienced interviewers. The response rate was 65.1%. The sample was nationally representative, except younger subjects were somewhat underrepresented. Of the 6646 baseline subjects, 140 subjects received a shortened version of the questionnaire, and as a consequence they did not receive questions about somatic disorders. Therefore, the number of subjects available at T_0_ was 6506 of which 386 subjects had MUS at T_0_. Of those 386, data were available of 324 subjects at T_1_. Those 324 subjects comprised our study cohort. They were assessed at T_0_ for the possible predicting factors for persistency of MUS, and reassessed for having MUS at T_1_. The attrition rate at 3 year follow-up for the 6,646 baseline subjects was 20.2%. The presence of psychopathology in the past 12 months at baseline was not significantly associated with attrition, after controlling for sociodemographic characteristics (34). The attrition rate for the 386 subjects with MUS at T_0_ was 16.1%; none of the variables presented in **Table 2** that were measured at T_0_ were statistically significant associated with this attrition. Also having MUS at T_0_ was not statistically significantly associated with attrition at T_1_.

### Variables

We use the following definition of MUS (33): presence of one or more physical symptom(s) in the past 12 months for which no adequate organ pathology or pathophysiological basis was found, and for which, according to the subject, a physician was consulted and/or medication was received, and which caused discomfort and functional impairment in the past 4 weeks as measured by the Short Form 36 (SF-36) (35). In line with the Somatic Symptom Disorder (SSD) definition in the *Diagnostic and Statistical Manual of Mental Disorders*, fifth edition (DSM-5) (36), we included the presence of discomfort and functional impairment in our definition. Persistence of MUS is defined as presence of MUS at both T_0_ and T_1_.

### Data sources/measurement

The measuring instruments for MUS and the possible predictors of persistent MUS are described in Table 1 [partially published in our previous studies (33, 53)]. Table 1 is available as an online addendum.

**Table 1:** Variables: definitions and measurement instruments (online addendum).

**Measurement**	**Measuring instrument**
**MEDICALLY UNEXPLAINED PHYSICAL SYMPTOMS**	
Subjects were considered to have MUS if their condition applied to both criteria mentioned below:	Face-to-face interview based on a questionnaire of physical symptoms, in which the main physical symptoms and chronic medical conditions of the CBS (Statistics Netherlands) questionnaire can be found.(37) These physical symptoms were based on self-report by the subjects during the interview, (38) and they had to specify if they sought medical treatment for that. Only in that case they were taken into account.
1. Presence of one of the following physical symptoms, experienced in the past 12 months, for which the subjects indicated that they visited a physician and/or received medication: a) Disturbing intestinal symptoms, existing longer than 3 months, for which no indication of an explanation existed (39–41). b) Back problems existing longer than 3 months, for which no indication of an explanation existed (40, 42). c) Other illness or physical symptoms that are long lasting (open question) and unexplained	All physical symptoms mentioned here (verbatim responses) were checked independently by two physicians (JES and CFC) to indicate whether or not they could be considered medically unexplained physical symptoms in general. If their judgments were not the same, they deliberated until consensus was achieved. We checked the answers on the open questions to see if an explanation was given about the intestinal symptoms, such as pancreatitis or hernia abdominalis, or the back problem, such as neck hernia or paraplegia. If this was the case, we did not include the subject in the unexplained group, but in the explained group. Examples of general symptoms that we considered to be medically unexplained physical symptoms are fibromyalgia, fatigue (such as chronic fatigue syndrome), pain without medical explanation (such as stress related pain in muscles), and physical symptoms accompanied with phrases such as “they can't find anything” or “if only I knew”.
2. Presence of limited functioning reported in the past 4 weeks, as indicated by two or more of the physical health subscales of the SF-36 (35, 43).	Interview based on the SF-36 physical health subscales: a) Physical functioning: some or severe limitations in at least one of the 10 items in this category b) Physical role functioning: any limitation reported in at least one of the four items in this category c) Bodily pain: pain leading to any limitation in normal work activities d) General health: describes mental or physical health as poor, and/or negative expectations about one's health.
Possible predicting factors of persistence of MUS	Face-to-face interview
Sex	Male/female
Partner status	Living with or without partner
Age	Based on the date of birth, divided in four age groups: a) 18–34 year b) 35–44 year c) 45–54 year d) 55–64 year age groups 18–24 years and 25–34 years were put together because of the small number of respondents per group; respectively *n* = 7 and *n* = 34.
Employment situation	Describes whether the subject has a paid job. Not only the main work situation was taken into account, but also any other type of work situations (e.g., a student who also has a payed employment, will be counted as having a paid job).
Education	Self-report: a) Primary, basic vocational or lower secondary b) Higher secondary c) Higher professional, University
Household income	Self-report: classification in based on the average income in the Netherlands in 2007 (€1,500 net income per month) a) Low: <average; ≤1,500 euro net per month b) Middle: 1 to 2 times average; 1,501–3,300 euro net per month c) High: more than 2 times average; >3,300 euro net per month
Childhood trauma before age 16	Self-report of emotional neglect (not listened to, ignored or unsupported), psychological abuse (yelled at, insulted, unjustly punished/treated, threatened, belittled, or blackmailed), physical abuse (kicked, hit, bitten or hurt with object or hot water), or sexual abuse (any unwanted sexual experience) before age 16. Emotional, psychological, or physical abuse was scored present if the respondent reported this had occurred more than once. Sexual abuse was also scored present if the respondent reported this had occurred once.
Parental psychopathology:	Lifetime mental problems parents. These questions were asked at T_1_.
Negative life events	The presence of 10 negative life events in the past 12 months, based on the “Brugha Life events section” (44)
Comorbid chronic medical disorder(s): Respiratory disorders (asthma, chronic obstructive pulmonary disease, chronic bronchitis, emphysema), cardiovascular disorders (severe heart disease, heart attack, hypertension, stroke), stomach or intestinal ulcers, severe intestinal symptoms (only if an explanation about the cause was given such as pancreatitis, hernia abdominalis), diabetes, thyroid disorder, chronic back pain (only if an explanation about the cause was given such as neck hernia, paraplegia, caused by accident), arthritis, migraine, cancer, impaired vision or hearing.	Interview based on questionnaire of physical symptoms, in which the main physical symptoms of the CBS (Statistics Netherlands) questionnaire can be found.(37) These physical symptoms were based on self-report by the subjects during the interview, and not by medical records (38). Comparisons between self-reports of chronic physical disorders and medical records show moderate to good concordance (45–47). Subjects were considered to have comorbid chronic medical disorder(s) if they reported to have been treated or monitored by a physician in the prior 12 months for one or more of the disorders, and after confirmation by two physicians (JES and CFC), in duplicate, that symptoms should be considered to be medically explained. The number of comorbid chronic medical disorders was counted.
DSM-IV mental disorders:DSM-IV mood disorder (major depression, dysthymia, bipolar disorder), anxiety disorder (panic disorder, agoraphobia (without panic disorder), social phobia, specific phobia, generalized anxiety disorder), and substance use disorder (alcohol/drug abuse and dependence). Here we combined the 12-month mood, anxiety, and substance use disorders, to form the group “any 12-month mental disorder”.	Composite International Diagnostic Interview Version 3.0 (CIDI 3.0) (38, 48, 49): The interviews were conducted by professional, experienced interviewers. Clinical calibration studies conducted in various countries have found that CIDI 3.0 (50) and earlier versions (51, 52) assess anxiety, mood and substance use disorders with generally good validity compared to blinded clinical reappraisal interviews.
Functioninga) Social role functioning b) Emotional role functioning c) Mental health d) Vitality e) Bodily pain f) Physical functioning g) Physical role functioning h) General health perceptions	Score (0–100) -low to high functioning- on the SF-36 sections: a) Social functioning scale b) Role-emotional scale c) Mental health scale d) Vitality scale e) Bodily pain f) Physical functioning g) Role-physical scale h) General health

### Bias

All definitions in Table 1 were predefined, and as strict as possible defined to support unambiguous interpretation. Definitions are in line with previously published papers using data from NEMESIS-2 (33, 53). Given that the data had already been collected by independent researchers, results could not be influenced by the authors. There were no missing data for most of the possible predictors of persistence at baseline. Only household income had 21 missings (6.5%) and the role-emotional scale of the SF-36 had one missing (0.3%).

### Quantitative variables and study size

Three hundred and twenty four subjects of the 386 subjects with MUS at T_0_ could be reassessment at T_1_. Those 324 were reassessed for having MUS at 3-year follow-up, and were assessed for the possible prediction factors for the persistency of MUS as described in Table 1.

### Statistical methods

All analyses were performed using STATA version 12.1. Given the relatively small group of subjects with MUS (i.e., 324 respondents), unweighted data were used. In order to describe the percentage that persisted in having MUS at follow-up, we performed a cross-tabulation of MUS-cases at T1. In order to define what risk factors at baseline influence persistency of MUS at follow-up (yes/no), we performed logistic regression analyses adjusted for sex and age (Table 2: model 1). In the next step, all significant variables adjusted for sex and age from model 1 were entered simultaneously in one more fully adjusted logistic regression analysis (Table 2: model 2), to find unconfounded estimates of risk factors for persistency. To test for linear trends, potential determinants (i.e., age, education level, and household income) were also modeled separately as continuous variables (P for trend test).

**Table 2:** Predictors of persistence of MUS (*N* = 324).

	**%**	**OR (95%CI)** **Model 1**	**OR (95%CI)** **Model 2**
**DEMOGRAPHIC VARIABLES**			
**SEX**			
Female	66.7	1.79 (1.08–2.97)^*^	1.23 (0.71,2.12)
**PARTNER STATUS**			
No partner	34.3	0.75 (0.46–1.23)
**AGE**			
18–34	13.3	1
35–44	21.6	1.26 (0.54–2.93)
45–54	30.2	2.04 (0.93–4.48)
55–64	34.9	1.81 (0.83–3.94)
p for trend		0.084
**EMPLOYMENT SITUATION**			
No paid job	42.3	1.65 (1.01–2.69)^*^	1.07 (0.63,1.82)
**EDUCATION**			
Primary, basic vocational or lower secondary	42.0	1.89 (1.03–3.48)^*^	1.80 (0.93,3.47)
Higher secondary	31.8	1.80 (0.95–3.41)	1.94 (0.99,3.81)
Higher professional or university	26.2	1	1
p for trend		0.053
**HOUSEHOLD INCOME**			
Low	32.4	1
Middle	50.8	0.70 (0.41–1.19)
High	16.8	0.55 (0.26–1.16)
p for trend		0.088
**VULNERABILITY**			
Any childhood trauma before age 16	41.1	1.24 (0.77–1.99)
Parental psychopathology: lifetime mental problems parents at T1	38.3	1.71 (1.05–2.78)^*^	2.01 (1.20,3.38)^*^
Any negative life event in the 12 months before baseline	66.1	0.74 (0.45–1.19)
**COMORBID PHYSICAL AND MENTAL DISORDERS**			
Number of comorbid chronic medical disorder(s)	Mean (se) 1.55 (0.09)	1.22 (1.05–1.42)^*^	1.19 (1.01,1.40)^*^
Any 12-month common mental disorder at baseline	28.7	1.47 (0.89–2.43)
Mental and physical functioning (scale 0–100)	Mean (se)	
Social role functioning	76.0 (1.44)	0.99 (0.98–1.00)^*^	1.00 (0.99,1.01)
Emotional role functioning	85.3 (1.80)	1.00 (0.99–1.01)
Mental health	78.7 (0.90)	1.00 (0.98–1.01)
Vitality	58.0 (1.11)	0.99 (0.98–1.00)
Bodily pain	54.1 (1.29)	0.98 (0.97–0.99)^*^	0.99 (0.98,1.00)
Physical functioning	66.9 (1.27)	0.98 (0.97–0.99)^*^	0.99 (0.97,1.00)^*^
Physical role functioning	44.3 (2.33)	0.99 (0.99–1.00)
General health perceptions	50.1 (1.09)	0.98 (0.97–0.99)^*^	0.99 (0.98,1.01)

Odds ratio (OR) with 95% Confidence Interval (CI) or mean with standard error (se), adjusted for sex and age (model 1), or adjusted for all variables in this column (model 2).

Model 1: adjusted for sex and age.

Model 2: adjusted for all variables in this column.

* *p < 0.05*.

## Results

### Outcome data and main results

Of the 324 subjects with MUS at baseline, 118 (36.4%) had persistent MUS, meaning they had MUS at both T_0_ and T_1_. Table 2 (model 1) shows that, in subjects with MUS at baseline, having persistent MUS is statistically significant predicted by female sex (OR 1.79; 95%CI 1.08–2.97), not having a paid job (OR 1.65; 95%CI 1.01–2.69), lower education (OR 1.89; 95%CI 1.03–3.48), parental psychopathology (1.71; 95%CI 1.05–2.78) and number of comorbid chronic medical disorder(s) (OR 1.22; 95%CI 1.05–1.42). A lower odds of having persistent MUS, is statistically significantly predicted by a higher score (meaning better functioning) on four scales of the SF-36. Those scales comprised social functioning (OR 0.99; 95%CI 0.98–10.00), bodily pain (0.98; 95%CI 0.97–0.99), physical functioning (0.98; 95%CI 0.97–0.99) and general health (0.98, 95%CI 0.97–0.99). The SF-36 subscales range from 0 to 100, the ORs refer to a 1-point increase of the subscales. The other variables shown in Table 2 did not statistically significantly predict persistency of MUS. Besides any 12-month mental disorder, persistency of MUS was also not statistically significant predicted by any 12-month mood disorder (OR 1.13; 95%CI 0.36–2.28) and any 12-month anxiety disorder (OR1.30; 95%CI 0.73–2.31) (not shown in table). Besides any childhood trauma before age 16, the subcategories (emotional neglect, psychological abuse, physical abuse, and sexual abuse) also did not statistically significant predicted persistence of MUS (not shown in table).

For the variables that were subdivided in three or more categories (age, education level, and household income) a p for trend was calculated. No statistically significant association was found with increasing age, education level, and household income and odds of having persistent MUS (see Table 2).

#### Multivariate analyses

A multivariate analysis was performed with the statistically significant findings (*p* < 0.05) from the analyses adjusted for sex and age. Of those, parental psychopathology (OR 2.01; 95%CI 1.20–3.38), number of comorbid chronic medical disorder(s) (OR 1.19; 95%CI 1.01–1.40) and the physical functioning scale of the SF-36 (OR 0.99; 95%CI 0.97–1.00) remained statistically significant predictors for having persistent MUS after 3 years. Female sex, not having a paid job, education level and social functioning, bodily pain and general health did not statistically significantly predict persistency of MUS anymore (Table 2; model 2).

## Discussion

### Key results and interpretation

To our knowledge, this study is among the first to explore the persistency of MUS in the adult general population. The findings are that over a 3-year follow-up, 36.4% of our sample with MUS at baseline had persistent MUS. Persistency of MUS is predicted by number of comorbid chronic medical disorder(s), lower education, female sex, parental psychopathology, and not having a paid job; in the logistic regression analysis adjusted for sex and age. A lower odds of having persistent MUS is predicted by better functioning as measured with the SF-36 in the areas social functioning, bodily pain, physical functioning, and general health. Our hypothesis about which variables predict persistency of MUS is confirmed -in the logistic regression analyses adjusted for sex and age (Table 2: model 1)-for number of comorbid chronic medical disorder(s), female sex, and partly for mental and physical functioning. These findings are in line with previous studies (16–19, 23, 54). An association between worse physical functioning at baseline measured with the SF-36 and persistent MUS at follow-up was found before in neurology patients (19). A possible explanation for the other variables that predicted persistency of MUS -namely lower education, parental psychopathology, and not having a paid job- is that they can have a negative influence on overall functioning and well-being (55–58).

Parental psychopathology had the highest OR in the multivariate logistic regression model, followed by number of comorbid chronic medical disorder(s). Also, better physical functioning measured by the SF-36 predicted persistent MUS statistically significant in this model. The other predictors found in the logistic regression analyses adjusted for sex and age (model 1) were not statistically significant in the more fully adjusted logistic regression analysis (model 2). Previous research showed an association between both paternal and maternal depression and increased risk of psychopathology in their offspring (59–61). Apparently, parental psychopathology is an indicator for vulnerability, which is also expressed in the increased risk of persistent MUS. This is in line with previous research about somatoform disorder in adolescents (27). The number of comorbid chronic medical disorder(s) as a predictor for persistence of MUS is in line with previous research (17, 18). A higher number of physical symptoms is also associated with a higher number of medical consultations (62). A possible explanation is, that it is more difficult to adequately cope with physical symptoms when they are partly MUS and partly due to a chronic medical disorder. A higher number of medical disorders only adds to this complexity (63).

In the less and more fully adjusted logistic regression analyses (model 1 and model 2), no statistically significant ORs were found for partner status, age, household income, any childhood trauma before age 16, any 12 months negative life event, any 12 month common mental disorder, and lower levels of functioning measured with the SF-36 in the subscales emotional role functioning, mental health, vitality and physical role functioning at baseline. This means our hypothesis about what variables predict persistency of MUS is not confirmed for childhood trauma, presence of negative life events, and presence of common mental disorders. Childhood adversities increase the risk of diverse chronic physical conditions -such as hypertension, heart disease and asthma- later in life (26, 64). Childhood adversities are also associated with the onset of mental disorders later in life (65), and childhood sexual abuse was found to be associated with MUS as well as chronic pelvic pain in previous research (66, 67). We have no explanation for the fact that we did not find similar results with regard to the persistency of MUS. We can hypothesize, however, that this finding indicates a difference between persistent MUS in the general population and the clinical setting. Perhaps childhood adversities are associated with more severe MUS or with help-seeking, which could explain the correlation between childhood adversities and MUS is more common in clinical settings compared to this general population research. Further research exploring the association between childhood adverse events and (explained or unexplained) physical symptoms is therefore warranted, taken into account the severity of the MUS and help-seeking behavior. MUS are associated with higher odds of having common mental disorders (33), but in this study we did not find a higher odds of having persistent MUS when common mental disorders were present at baseline.

Research performed in general hospital and in primary care showed persistency rates of 24–54% (1, 16–19), with follow-up times varying from 8 to 15 months. In the general population, persistence MUS was found in 33.6% of MUS-pain, with an 11 year follow-up (28). The percentage of persistency we found in this study, 36.4%, does not differ largely from the persistency rates found in the majority of the beforementioned studies. However, the follow-up period varies between the studies, which makes it difficult to compare these persistency rates. We expected higher persistency rates when follow up periods are shorter, but when we compare these studies, there is no clear relation between length of follow-up and persistency rate.

## Limitations

We used an existing longitudinal epidemiological database that was aimed at assessing mental disorders. This database also contains information about physical symptoms and possible predictors of mental disorders. For the current study, we were therefore limited to the available data. The information given by the respondents was used to determine whether or not the physical symptoms could be considered MUS. For this purpose, all physical symptoms mentioned were checked independently in duplicate by two physicians (JES and CFC). We did not have access to the medical records of the respondents. Comparisons between chronic medical conditions and symptoms as assessed by medical records show moderate to good concordance (46, 68–70) Currently, the focus is more on the way patients cope emotionally, cognitively and behaviorally with their physical symptoms, then whether they are explained or not (36). We included the presence of discomfort and functional impairment in our definition of MUS, in order to approach the criteria of the Somatic Symptom Disorder as described in the DSM 5 (36). Although we used an existing database, we believe our methods of operationalization and classification are reasonable for MUS. We only classified symptoms that led to treatment seeking as MUS, because this is an indication that the complaints are not quickly passing symptoms that are not severe enough to be considered MUS (as in line with our definition). The definition of MUS is based on the older concept of somatization (71). Somatization is defined by Lipowski as “a tendency to experience and communicate somatic distress in response to psychosocial stress and to seek medical help for it” (72). Seeking treatment is an essential part of the definition somatisation and MUS (72–74), so in our article we stay in line with this. It should be noted however, that the definition of MUS remains subject of debate (75, 76).

A limitation of the current study is that we do not know the onset date of the MUS, we only know whether or not the MUS were present at T_0_, but and not for how long already. Due to the limited available variables, we were not able to explore health anxiety and avoidance behavior. The power of different predictor variables can also differ because of statistical features of the variable (e.g., binary vs. continuous variables and reliability of assessments). It is also possible that limited power has led to non-significant correlations in model 2, which means larger study samples are needed to reassess this.

### Generalizability

NEMESIS-2 is a large, nationally representative sample of the adult Dutch general population. Therefore, the results of this study can be extrapolated to the general population of the Netherlands, and possibly to more countries. We did not assess the onset date of the MUS, therefore the MUS at baseline will be a mixture of first-onset symptoms and chronic symptoms. This limits the generalizability to unselected groups of persons with MUS, given that the role of predictor variables can vary between groups with first-onset symptoms and groups with chronic symptoms.

## Conclusion

In this general population study, MUS are persistent in 36.4% measured over a 3-year interval. Persistency is significantly predicted by parental psychopathology and having comorbid chronic medical disorder(s). The parental psychopathology is an interesting subject for further research, given that it can possibly lead to early interventions and specific treatment options. For example, when the stepped care approach as described in the Dutch guideline for treatment of MUS (6) is used, collaboration with or referral to a physiotherapist or psychologist could be recommended in an early stage for persons with a high risk of having persistent MUS.

## Author contributions

JE, MtH, RdG, CR, HvW, and CvF-C: designed the study; RdG and MtH: collected the data; JE and CvF-C: checked data; MtH: performed the analyses with input of JE, RdG, and CvF-C. JE took the lead in writing the manuscript. All authors contributed to the interpretation of the results, provided critical feedback and contributed to shaping the research analysis and manuscript.

### Conflict of interest statement

The authors declare that the research was conducted in the absence of any commercial or financial relationships that could be construed as a potential conflict of interest.
